# Mass Spectrometry Detection and Imaging of a Non‐Covalent Protein–Drug Complex in Tissue from Orally Dosed Rats

**DOI:** 10.1002/ange.202202075

**Published:** 2022-07-13

**Authors:** Eva Illes‐Toth, Oliver J. Hale, James W. Hughes, Nicole Strittmatter, Jonathan Rose, Ben Clayton, Rebecca Sargeant, Stewart Jones, Andreas Dannhorn, Richard J. A. Goodwin, Helen J. Cooper

**Affiliations:** ^1^ School of Biosciences University of Birmingham Edgbaston Birmingham B15 2TT UK; ^2^ Imaging & Data Analytics Clinical Pharmacology & Safety Sciences Biopharmaceuticals R&D, AstraZeneca Cambridge CB4 0WG UK; ^3^ Animal Sciences & Technologies Clinical Pharmacology & Safety Sciences, AstraZeneca Babraham Research Campus Babraham Cambridge, CB22 3AT UK

**Keywords:** Ex vivo Tissue, Mass Spectrometry Imaging, Native Ambient Mass Spectrometry, Protein–Drug Complex

## Abstract

Here, we demonstrate detection by mass spectrometry of an intact protein–drug complex directly from liver tissue from rats that had been orally dosed with the drug. The protein–drug complex comprised fatty acid binding protein 1, FABP1, non‐covalently bound to the small molecule therapeutic bezafibrate. Moreover, we demonstrate spatial mapping of the [FABP1+bezafibrate] complex across a thin section of liver by targeted mass spectrometry imaging. This work is the first demonstration of *in situ* mass spectrometry analysis of a non‐covalent protein–drug complex formed *in vivo* and has implications for early stage drug discovery by providing a route to target‐drug characterization directly from the physiological environment.

## Graphical abstract

The intact protein–drug complex formed in vivo between endogenous liver fatty acid binding protein (FABP1) and the small molecule therapeutic bezafibrate was detected in ex vivo liver tissue from orally dosed rats by native ambient mass spectrometry. The spatial distribution of the protein–drug complex was imaged by nanospray desorption electrospray ionization.

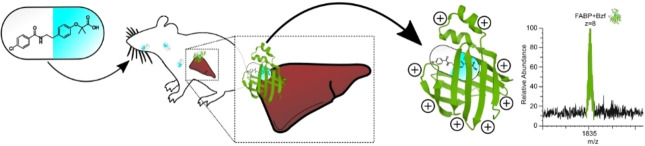

Native ambient mass spectrometry enables the analysis of folded proteins directly from thin tissue sections, providing simultaneous information on protein structure and spatial distribution. We have previously demonstrated the detection of endogenous protein assemblies from thin tissue sections of kidney, liver and brain.^[1]^ Here, we apply native ambient mass spectrometry to the analysis of a protein–drug complex formed *in vivo* following oral dosing of rats. The stoichiometry of the complex, the variation in its relative abundance over time and its spatial distribution in liver tissue were determined.

The fatty acid binding proteins are a family of ≈15 kDa proteins that are responsible for regulating long‐chain fatty acid metabolism and transport.^[2]^ Liver fatty acid binding protein (FABP1) is a particularly abundant protein constituting up to 5 % of cytosolic proteins in the liver.^[3]^ Its structure comprises ten antiparallel β‐strands creating a β‐barrel with a prominent inner cavity, which forms the lipid binding site.^[4]^ Unlike other FABPs, FABP1 binds two fatty acids per protein molecule, with the binding affinity of the second ligand ≈100‐fold lower than that of the first.^[5]^ FABP1 is involved in the transport of various lipid‐lowering drugs, the peroxisome proliferators, from the cytosol to the nucleus where they interact with the peroxisome proliferator‐activated receptor α.[^5^, ^6^] Bezafibrate (Figure S1, Supporting Information) is one such drug. It is unusual amongst the fibrates in that it binds to FABP1 with a 1 : 1 stoichiometry and that binding occurs at the lower affinity site.^[7]^ The binding affinity of bezafibrate to rat FABP1, as determined by fluorescence measurements, is ≈45 μM. The absorption of bezafibrate is complete and rapid, and its clearance is fast, with a half life of 2.1 hours (serum) in humans^[8]^ and 4–5 hours (plasma) in rats.^[9]^

In this work, we demonstrate that the intact non‐covalent protein‐drug complex formed *in vivo* between endogenous FAPB1 and orally dosed bezafibrate can be detected directly from *ex vivo* liver tissue by native ambient mass spectrometry. Han‐Wistar rats were orally dosed with bezafibrate and euthanized either 2 hours or 6 hours post‐dose (see Table S1, Supporting Information). Livers were dissected and sectioned at 10 μm thickness. The tissue sections were analyzed by native liquid extraction surface analysis mass spectrometry (LESA MS)^[10]^ and the protein–drug complex was detected. Targeted mass spectrometry imaging (MSI) in which native nanospray desorption electrospray ionization (nano‐DESI)^[11]^ was coupled with proton transfer charge reduction (PTCR)^[12]^ enabled the spatial distribution of the protein‐drug complex within the tissue section to be visualized. Typically, LESA offers higher sensitivity and greater nanoelectrospray stability than nano‐DESI, whereas nano‐DESI offers greater spatial resolution for mass spectrometry imaging.

Representative full‐scan native LESA mass spectra obtained from thin liver sections of control and dosed animals are shown in Figure S2, Supporting Information. Unbound FABP1 was detected in all tissues and the identity of the protein was confirmed by top‐down LESA mass spectrometry, in which the intact protein ion is fragmented and the mass‐to‐charge (*m/z*) ratios of the product ions are searched against theoretical *m/z* values derived from a protein database (Figure  S3, Supporting Information). A number of proteins, including acyl‐coA binding protein (ACBP) and thymosin‐β4, were detected in addition to FABP1 (see Figures S4–S7, Supporting Information). The [FABP1+bezafibrate] complex is not detected in these full scan mass spectra; however, the presence of bezafibrate in the dosed tissue is confirmed by examination of the low *m/z* region (Figures S8 and S9, Supporting Information). Peaks corresponding to protonated, sodiated and potassiated bezafibrate were observed in the mass spectra from dosed tissue but were not observed in the control.

In order to detect the [FABP1+bezafibrate] complex, it was necessary to perform targeted analyses in the selected ion monitoring (SIM) mode in which data are collected over a narrow *m/z* range. This approach enables accumulation of low abundance ions and ameliorates ion suppression effects. Figure 1a–f show representative SIM‐mode mass spectra obtained following LESA of liver sections of each of the dosed and control animals. Replicate SIM‐mode mass spectra from liver sampled at three separate locations are shown in Figure S10, Supporting Information. These mass spectra reveal peaks corresponding to 8+ ions of the [FABP1+bezafibrate] complex in the dosed samples. These peaks are not detected in the control (undosed) samples. The abundance of the [FABP1+bezafibrate] complex is greater in the samples taken 2 hours post‐dose than in those taken 6 hours post‐dose. This observation is consistent with the known rapid excretion of the drug.^[9]^

**Figure 1:**
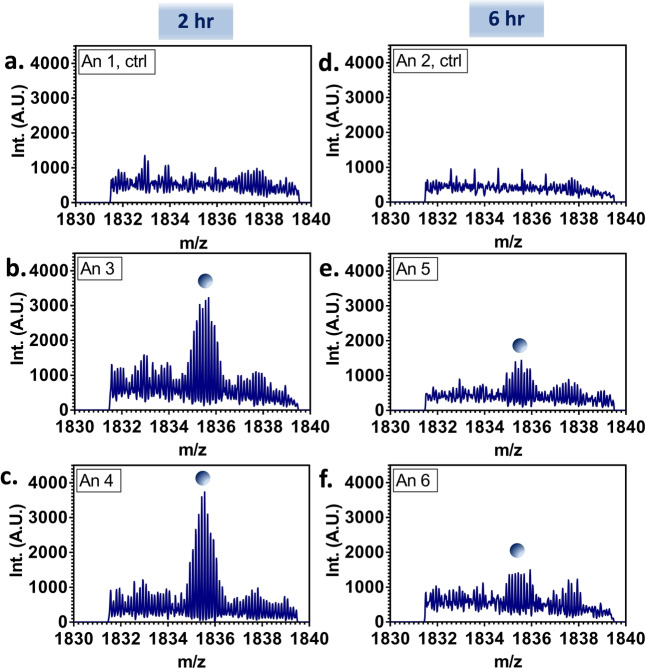
Native LESA SIM‐mode mass spectra obtained from rat liver sections. The peak indicated with the blue circle corresponds to the [FABP1‐bezafibrate] complex in the 8+ charge state (i.e., [FABP1+bezafibrate+8H^+^]^8+^). The complex was not observed in the control tissue (a and d, Animals 1 and 2), and was observed in higher abundance in the tissue taken 2 hours post‐dose (b and c, Animals 3 and 4) than in that taken 6 hours post‐dose (e and f, Animals 5 and 6).

To confirm the identity of the ligand, tandem mass spectrometry was performed on a modified Thermo Orbitrap QE‐HF mass spectrometer. The 8+ ions corresponding to the putative [FABP1+bezafibrate] complex were isolated and subjected to fragmentation by higher‐energy collision dissociation (HCD). The results are shown in Figure 2a–h and reveal that the complex dissociates to the free protein, also observed in the 8+ charge state (i.e., a mass loss of 361 Da was observed). Peaks corresponding to bezafibrate in the low *m/z* region were not observed. These observations suggest that the bezafibrate leaves as a neutral molecule, which is unusual but not without precedent.^[13]^

**Figure 2:**
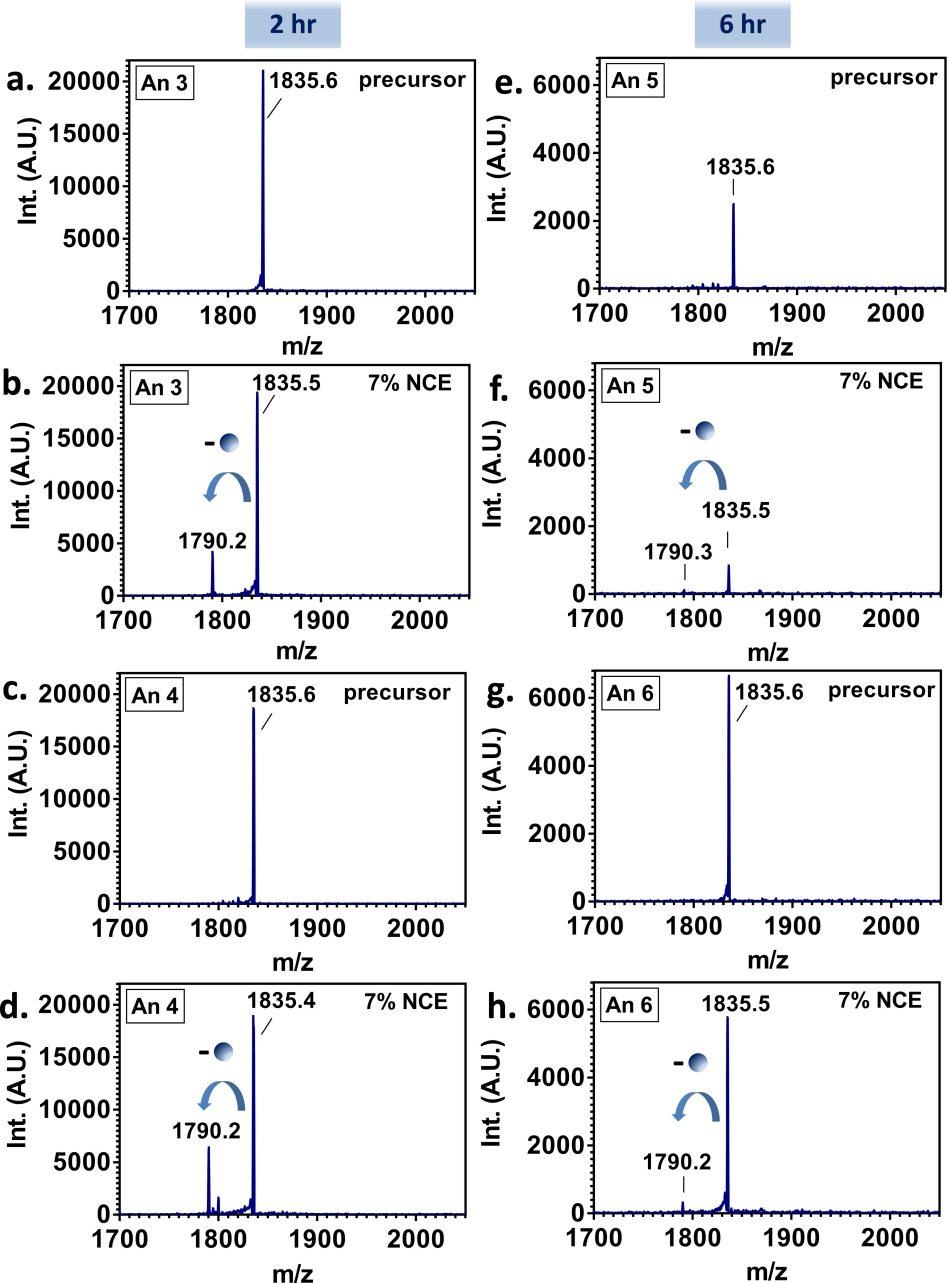
Native LESA HCD MS/MS of [FABP1+bezafibrate] ions. Precursor ions (*m/z* 1835; 8+ charge state) were isolated and fragmented by HCD at 7 % normalized collision energy (NCE). Panels a–d show the results from samples taken 2 hours post‐dose (Animals 3 and 4); panels e–h show results from samples taken 6 hours post‐dose (Animals 5 and 6). The blue arrows indicate the release of the drug corresponding to the loss of bezafibrate as a neutral ligand.

Lastly, we performed mass spectrometry imaging of a dosed liver section by use of native nanospray desorption electrospray ionization (nano‐DESI). Nano‐DESI is less well‐established than the more mainstream imaging techniques of matrix assisted laser desorption ionisation (MALDI)^[14]^ and desorption electrospray ionisation (DESI).^[15]^ Although MALDI and DESI have been applied to protein imaging under denaturing conditions,^[16]^ to date only LESA and nano‐DESI have been demonstrated for native mass spectrometry imaging.[^1c^, ^1d^, ^17^] Nano‐DESI offers higher spatial resolution than LESA. In this work, we harnessed a targeted proton transfer charge reduction (tPTCR) strategy within the nano‐DESI workflow to improve signal specificity and sensitivity. Proton transfer charge reduction (PTCR) involves transfer of protons from a protein cation to a reagent anion resulting in charge reduction of the protein cation. PTCR is particularly useful for native ambient mass spectrometry as it can confirm the presence of low abundance protein ions and can provide intact mass information from low resolution mass spectrometry,^[1c]^ see Figure S11, Supporting Information. The tPTCR strategy involves multiplexed serial precursor ion selection and storage of a range of ions in the ion routing multipole followed by simultaneous PTCR of the entire cohort. Ion images are then generated from product (i.e., charge‐reduced) ions only to avoid contribution from any background ions which may overlap with the precursor. Here, the target precursor ions comprise unbound FABP1 (8+ charge state), the heme‐bound α‐globin monomer (8+ charge state), ACBP (6+ charge state) in addition to the [FABP1+bezafibrate] complex (8+ charge state), see Table S2, Supporting Information. Figure S12, Supporting Information, shows a representative nano‐DESI tPTCR mass spectrum obtained from a single line scan within the imaging dataset. The detection of the charge‐reduced product ions confirms the presence of the series of proteins. Figure 3a shows an optical image of the liver tissue region. Figure 3b–d and Figure S13, Supporting Information, show images obtained from the tPTCR nano‐DESI experiment. The ion images for the [FABP1+bezafibrate] complex reveal it to be distributed throughout the bulk tissue and absent in the blood vessel. For contrast, ion images for heme‐bound α‐globin, a sub‐unit of haemoglobin, are shown. As expected, heme‐bound α‐globin is abundant in the blood vessel and absent from the bulk tissue.

**Figure 3:**
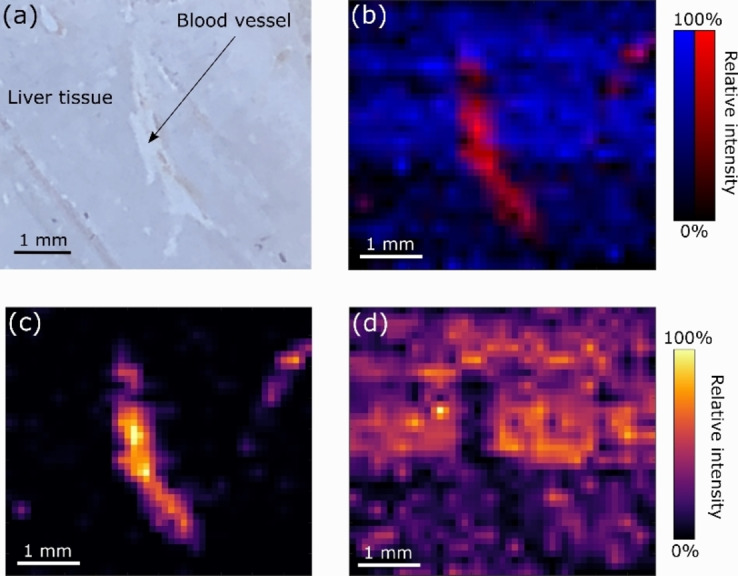
Targeted proton transfer charge reduction (tPTCR) nano‐DESI mass spectrometry imaging of liver tissue from orally dosed rat (Animal 3). a) optical image of a blood vessel within liver tissue. b) Composite ion image of charge‐reduced heme‐bound α‐globin (7+ and 6+ charge states; *m/z* 2259.9 and *m/z* 2636.3 respectively, red) and the charged‐reduced [FABP+bezafibrate] complex (7+ and 6+ charge states; *m/z* 2097.5 and *m/z* 2446.9 respectively, blue). c) Ion image composed from charge‐reduced heme‐bound α‐globin (7+ and 6+ charge states) showing abundance in blood vessels. d) Ion image composed from charge‐reduced [FABP+bezafibrate] complex (7+ and 6+ charge states) showing abundance in bulk tissue and absence in the blood vessel.

The results presented constitute the first demonstration of in situ analysis of an intact protein‐drug complex formed *in vivo*. Firstly, we used liquid extraction surface analysis mass spectrometry to detect the complex formed between endogenous FABP1 and orally dosed bezafibrate directly from rat liver tissue. The free protein and free drug were both detected in the full scan mass spectra, together with a number of other proteins. Protein identities were confirmed on the basis of tandem mass spectrometry. It was necessary to perform SIM‐mode mass spectrometry, which acquires data over a focused *m/z* range and thereby addresses ion suppression, dynamic range and space charge effects, to detect the complex. That is, by reducing the *m/z* range, it is possible to accumulate low abundance ions without compromising mass spectrometer performance. The abundance of the [FABP1+bezafibrate] complex was greater in the samples taken 2 hours post‐dose than in those taken 6 hours post‐dose consistent with excretion of the drug. To confirm the identity of the [FABP1+bezafibrate] complex, tandem mass spectrometry was performed. The results showed that collisional activation of the putative complex precursor ions in the 8+ charge state resulted in loss of the neutral ligand to give the free FABP1 protein, also in the 8+ charge state. This fragmentation pattern is unusual—typically a ligand will dissociate from a complex in a charged state—but has been observed in some cases. Together, the LESA SIM‐mode mass spectra acquired on the Orbitrap Eclipse and the LESA HCD tandem mass spectra acquired on the modified QE‐HF mass spectrometer, confirm the presence of the [FABP1+bezafibrate] complex in the tissue. The benefit of these native ambient mass spectrometry approaches is that structural information is retained in the gas‐phase. Potential future applications include determining stoichiometry and sites of binding in drug discovery. It may also be possible to improve sensitivity by combining native LESA extraction with native liquid chromatography MS.^[18]^

We also introduce the concept of targeted PTCR for mass spectrometry imaging of proteins. The tPTCR approach addresses some key challenges for imaging of low abundance proteins, including the potential for interference from overlapping background signals and need to obtain intact mass information from low resolution mass spectrometry data. tPTCR is similar in principle to multiple reaction monitoring in which the presence of a precursor is confirmed on the basis of a defined set of product ions. By generating images for product (charge‐reduced) ions only, any contributions from non‐specific signals which overlap with the precursor *m/z* are eliminated. In addition, the unique set of *m/z* values corresponding to the series of charge‐reduced product ions allows deconvolution of a protein's intact mass without the need for high resolution mass spectrometry (whose timescale is generally incompatible with mass spectrometry imaging). In this work, the nano‐DESI tPTCR approach enabled visualization of the distribution of the [FABP1+bezafibrate] complex throughout the bulk liver tissue and the heme‐bound α‐globin in the vasculature. The precursor ions selected for tPTCR all had *m/z* <2000, and this is a limitation of the approach currently. The Orbitrap Eclipse mass spectrometer used in this work enables precursor selection via a quadrupole or a linear ion trap. Multiplexed precursor selection is only possible by use of the quadrupole which has an upper *m/z* limit for isolation of 2000 (compared with 8000 for the linear ion trap). Further development of tPTCR mass spectrometry imaging would require either a higher *m/z* range quadrupole or the facility for multiplexing with the ion trap.

## Conflict of interest

The authors declare no conflict of interest.

## Supporting information

As a service to our authors and readers, this journal provides supporting information supplied by the authors. Such materials are peer reviewed and may be re‐organized for online delivery, but are not copy‐edited or typeset. Technical support issues arising from supporting information (other than missing files) should be addressed to the authors.

Supporting Information

## Data Availability

Data supporting this work is available from https://doi.org/10.25500/edata.bham.00000845.
